# *Magnaporthe oryzae* systemic defense trigger 1 (MoSDT1)-mediated metabolites regulate defense response in Rice

**DOI:** 10.1186/s12870-020-02821-6

**Published:** 2021-01-11

**Authors:** Guihua Duan, Chunqin Li, Yanfang Liu, Xiaoqing Ma, Qiong Luo, Jing Yang

**Affiliations:** 1 State Key Laboratory for Conservation and Utilization of Bio-Resources in Yunnan, Yunnan Agricultural University, Kunming, 650201 China; 2 Key Laboratory of Agro-Biodiversity and Pest Management of Ministry of Education, Yunnan Agricultural University, Kunming, 650201 China; 3 Quality Standard and Testing Technology Research Institute, Yunnan Academy of Agricultural Sciences, Kunming, 650205 China

**Keywords:** Rice, *Magnaporthe oryzae*, Metabolites, Effectors, Defense response

## Abstract

**Background:**

Some of the pathogenic effector proteins play an active role in stimulating the plant defense system to strengthen plant resistance.

**Results:**

In this study, ultra-high performance liquid chromatography-quadrupole time-of-flight mass spectrometry (UHPLC/Q-TOF-MS) was implemented to identify altered metabolites in transgenic rice containing over-expressed *M. oryzae* Systemic Defense Trigger 1 (MoSDT1) that was infected at three-time points. The characterized dominating metabolites were organic acids and their derivatives, organic oxygen compounds, lipids, and lipid-like molecules. Among the identified metabolites, shikimate, galactinol, trehalose, D-mannose, linolenic acid, dopamine, tyramine, and L-glutamine are precursors for the synthesis of many secondary defense metabolites Carbohydrate, as well as amino acid metabolic, pathways were revealed to be involved in plant defense responses and resistance strengthening.

**Conclusion:**

The increasing salicylic acid (SA) and jasmonic acid (JA) content enhanced interactions between JA synthesis/signaling gene, SA synthesis/receptor gene, raffinose/fructose/sucrose synthase gene, and cell wall-related genes all contribute to defense response in rice. The symptoms of rice after *M. oryzae* infection were significantly alleviated when treated with six identified metabolites, i.e., galactol, tyramine, L-glutamine, L-tryptophan, α-terpinene, and dopamine for 72 h exogenously. Therefore, these metabolites could be utilized as an optimal metabolic marker for *M. oryzae* defense.

**Supplementary Information:**

The online version contains supplementary material available at 10.1186/s12870-020-02821-6.

## Background

Plant pathogens exploit divergent mechanisms to infect plants; however, all strategies require the release of effector molecules into plant cells to initiate a successful infection and colonization. On the other way round, it has been well documented that the plants take two paths of immune defense responses to react against the virulent infections [1]. The first defense mechanism is pathogen-associated molecular patterns/microbe-associated molecular pattern (PAMP/MAMP)-triggered downstream genes that cause asymptomatic or non-small H responses, which are PAMP pattern-triggered immunity (PTI) or non-host resistance [2–4]. The second defense mechanism is the pathogenic effector protein triggered downstream genes, which result in a race-specific H response and it is effector-triggered immunity (ETI) or vertical resistance [3, 5]. However, the understanding of underlying biochemical mechanisms and genetic backgrounds of plant resistance remains elusive. Also, the biochemical mechanism-based strategies to precisely prevent and control plant disease have not been reported.

Metabolic signaling in plants is crucial in both physiological as well as biochemical infection resistance system, making it a promising direction of enhancing plant disease resistance [6]. Metabolites produced by plants are the most direct products responding to external stresses. And these direct products are subsequently reflected by symptomatic phenotypes of plants under stress state. Carbohydrates and amino acids are the primary metabolites responsible to combat the stress in plants [7]. When pathogenic fungi infect plants, sugar levels in plant tissues elevate to activate plant defense responses, which participate in regulating hormonal signaling network of plant immune system and induce plants to present stronger resistance to pathogens [8]. Diverse organic oxygen compounds [9], lipids [10], benzenoids [11], and organic acids [12] play an important role in plant defense responses. Critical metabolic pathways, such as carbohydrate metabolism, amino acid metabolism, galactose metabolism, glyoxylate, and dicarboxylate metabolism, starch and sucrose metabolism, ABC transporters, etc., are activated when plants are infected by pathogens [8]. Therefore, the understanding of certain related pathways in carbohydrate metabolism, amino acid metabolism, and ABC transporters could provide a provoking insight for a more specific genetic modification platform to strengthen plant resistance systems.

The application of metabolomics in studying interactions between plants and pathogens has achieved significant progress [13]. By performing metabolomic analysis, certain metabolites could be identified and employed as the potential biomarkers during infection responses by *Phytophthora infestans* in tomato [14]. By applying synthetic precursors for some important secondary defense metabolites, such as phenylalanine, allantoin, glutamate, thiamine, glycine, serine, asparagine, trehalose, etc., plants not only achieve growth but also boost resistance [15]. Based on the metabolomic data, the selection of crucial metabolites as exogenous treatment of plants is conducive to the growth and resistance of plants, which is beneficial to environmental protection of farmland and low/zero pesticides as well. Hence, exogenous metabolites have great potential for application in environmental control of plant diseases. It has been reported that transgenic plants with heterologous expression of effector gene can be used as an ideal model for identifying effective plant resistance resources. The mechanism of plant resistance enhancement at the molecular level has already been standardized [16, 17], but there are no reports available that deciphers the biochemical mechanism for improving the resistance. In a previous report, it was found that the growth of MoSDT1-transgenic rice was approximately comparable with that of wild-type rice; however, the resistance against *M. oryzae* was significantly improved in MoSDT1-transgenic rice [18]. UHPLC-Q-TOF MS was utilized to identify the metabolites and metabolic pathway alterations in *M. oryzae* infected MoSDT1-transgenic rice at different time points, characterize key metabolites that were released in response to the infection, and analyzed leading metabolite expressions of synthetic genes along with defense-related genes in MoSDT1-transgenic rice. We employed standard products as alternatives of defensive metabolites to treat wild-type rice (it is to pre-inoculate rice with *M. oryzae* and then implement spray treatment with atomized compounds after 72 h), then recorded the symptoms of *M. oryzae*-infected rice and determined the role of these key defense metabolites in elevating resistance in rice. The present study reveals the mechanism at the biochemical level for improving the resistance of MoSDT1-transgenic rice infected with *M. oryzae* and provides fundamental data for the prevention of plant diseases in the future.

## Results

### Differential metabolites identified from MoSDT1-transgenic line inoculated with blast strain

Primary metabolites of *M. oryzae*-infected MoSDT1*-*transgenic rice at 0 h, 72 h, and 120 h were identified by UHPLC-Q-TOF MS. Compared with *M. oryzae*-infected wild type rice, 154 differential metabolites were identified in MoSDT1*-*transgenic rice when infected with *M. oryzae* at the time point of 0 h (Supplementary Table 1). Among these, 95 were up-regulated, 22 were organic acids and their derivatives, 17 were organic oxygen compounds, 6 lipids and lipid-like molecules, and 32 were unclassified (Table 1; Supplementary file 1). On the other hand, 59 were down-regulated metabolites, of which 3 were organic oxygen compounds, 2 organic acids and derivatives, 3 lipids and lipid-like molecules, 49 were uncategorized (Table 1; Supplementary file 1). After 72 h of incubation, 138 differential metabolites were identified (Supplementary Table 1), 95 of which were up-regulated, 16 organic oxygen compounds, 14 organic acids and derivatives, 10 nucleotides and analogs, 8 lipids and lipid-like molecules, 5 benzenoids, and 32 unclassified (Table 1; Supplementary file 1). Of the 43 down-regulated metabolites, 10 were organic acids and derivatives, 5 organic oxygen compounds, 4 organic heterocyclic compounds, and 18 metabolites were not classified (Table 1; Supplementary file 1). In total, 110 differential metabolites (Supplementary Table 1) and 68 up-regulated metabolites were identified at 120 h, which included 18 organic oxygen compounds, 15 organic acids and derivatives, 10 lipids and lipid-like molecules, 15 unclassified (Table 1; Supplementary file 1). Further, 42 metabolites were down-regulated, including 6 lipids and lipid-like molecules, 6 organic acids and derivatives, and 16 unclassified (Table 1; Supplementary file 1). In this study, up-regulated metabolites were identified at three time points (0 h, 72 h, and 120 h). The MoSDT1*-*transgenic rice infected with *M. oryzae* showed mainly organic acids and corresponding derivatives, organic oxygen compounds, lipids and lipid-like molecules. It is in accordance with a previous study that also found primary metabolic alterations, for example, in amino acids, organic acid, and carbohydrates, in plants resistant to *Bipolaris oryzae*, *M. oryzae* etc. [19]. Moreover, the results were consistent with the defense response metabolites up-regulated by *Rhizoctonia solani* infecting rice [9].

**Table 1:** Number of differential metabolites belonging to super class

Super class	Mo10 vs WT (0 h)		Mo10 vs WT (72 h)		Mo10 vs WT (120 h)	
	Increased	Decreased	Increased	Decreased	Increased	Decreased
Organic acids and derivatives	22	2	14	10	15	6
Lipids and lipid-like molecules	6	3	8	3	10	6
Organoheterocyclic compounds	6	1	7	4	5	4
Organic oxygen compounds	17	3	16	5	18	2
Organonitrogen compounds	1	0	1	0	0	2
Nucleosides, nucleotides, and analogues	7	1	10	2	3	1
Lignans, neolignans and related compounds	1	0	1	0	1	0
Benzenoids	3	0	5	1	2	2
Phenylpropanoids and polyketides	1	2	2	1	0	3
N/A	31	47	31	17	14	16
Total	95	59	95	43	68	42

### Analysis of main defense metabolites between MoSDT1-transgenic line challenged with blast strain

The study analyzed the differential metabolites that responded to *M. oryzae* infection in MoSDT1-transgenic rice at 0 h, 72 h, and 120 h (Supplementary Table 1). Galactinol, D-mannose, shikimate, glyceric acid, and sucrose belonging to organic oxygen compounds, L-glutamine and L-tryptophan, which belong to organic acids and derivatives, salicylic acid among benzenoids, unclassified D-biotin, and camptothecin metabolites, all showed significant accumulation at the three time points (Supplementary Table 1). Linolenic acid and linoleic acid among lipids and lipid-like molecules, raffinose among organic oxygen compounds, tyramine among benzenoids, and unclassified alpha-pinene showed significant accumulation at 72 h. Oxalate among organic acids and derivatives decreased at 72 h and became undetectable at the other two time points. Anthranilic acid and dopamine in benzenoids were accumulated significantly at 0 h and 120 h. Organic oxygen compounds [9], lipids and lipid-like molecules [10], benzenoids [11, 12], and many unclassified metabolites were detected in *M. oryzae*-infected MoSDT1-transgenic resistant rice. These are the core defense metabolites in *M. oryzae*-infected MoSDT1-transgenic plants.

### Analysis of enriched pathways between MoSDT1-transgenic line challenged with blast strain

At 0 h in the *M. oryzae* infected MoSDT1-transgenic rice, KEGG enriched differential metabolites included 22 pathways, 7 of them belong to amino acid metabolic pathways (Supplementary Table 2), 10 belong to carbohydrate metabolic pathway (Supplementary Table 3), and 4 of them belong to nuclear metabolic pathways (Supplementary Table 4) and ABC transporters (Supplementary Table 4). At 72 h, the differential metabolites enriched KEGG mainly consists of 15 pathways, among which 5 belong to amino acid metabolic pathway (Supplementary Table 2), 7 belong to carbohydrate metabolic pathway (Supplementary Table 3), and 2 belong to nitrogen metabolic pathway (Supplementary Table 4) and ABC transporters (Supplementary Table 4). At 120 h, there were 12 differential metabolite enriched pathways, among which only one belongs to amino acid metabolic pathway (Supplementary Table 2), 7 belong to carbohydrate metabolic pathway (Supplementary Table 3), and 2 belong to nitrogen metabolism (Supplementary Table 4) and ABC transporters (Supplementary Table 4). More differential metabolites were involved in the main KEGG pathway between MoSDT1-transgenic line inoculated with or without blast strain (Supplementary Table 5). The result indicated that these metabolic pathways were activated during the infection process. Suharti et al. (2016) reported that carbohydrate metabolic pathway and amino acid metabolic pathway were the main pathways being regulated when resistant rice was infected with *Rhizoctonia solani* [9]. In this study, these two pathways were also identified at three time points after analyzing differential metabolites KEGG enrichment, which further confirmed the increased resistance in MoSDT1-transgenic rice.

### Analysis of metabolic defense response/synthetic gene expression in *M. oryzae*-infected MoSDT1-transgenic rice

**Fig. 1:**
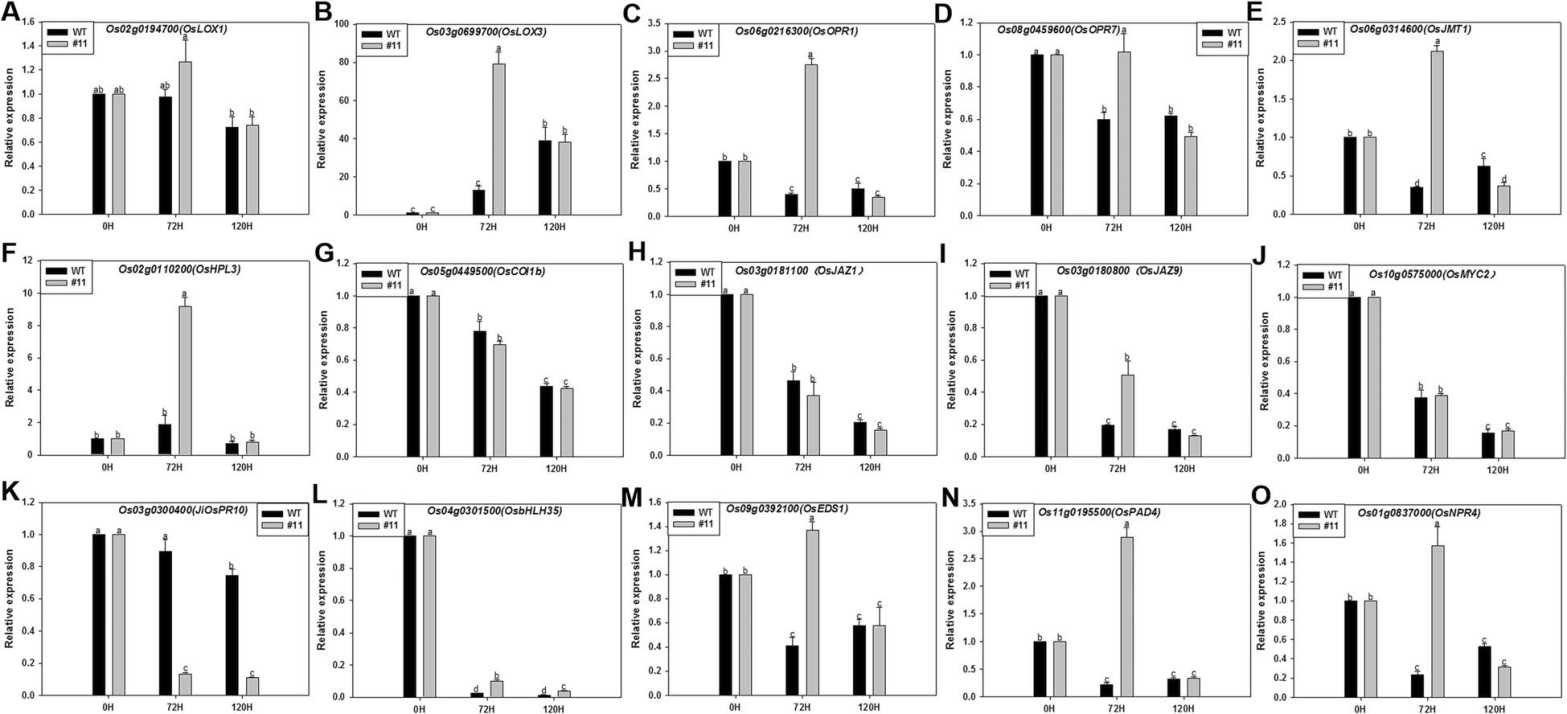
Expression of jasmonic acids synthesis/responsive pathway genes and salicylic acid synthesis/receptor genes in MoSDT1-transgenic line inoculated with rice blast strain at 0 h, 72 h, and 120 h. Relative expression levels of jasmonic acids synthesis/responsive pathway genes, namely, *OsLOX1/3* (**a**, **b**), *OsOPR1/7* (**c**, **d**), *OsJMT1* (**e**), *OsHPL3* (**f**), *OsCOI1b* (**g**), *OsJAZ1* (**h**), *OsJAZ9* (**i**), *OsMYC2* (**j**), *JiOsPR10* (**k**), and *OsbHLH35* (**l**), are shown. Salicylic acid synthesis/receptor genes of *OsEDS1/OsPAD4* (**m**, **n**) and *OsNPR4* (**o**) are shown. Different letters (a, MoSDT1-transgenic rice line, differential metabolites at three time points: b, 0 h; c, 72 h; d, 120 h) with a representative significant difference as *P* < 0.05

**Fig. 2:**
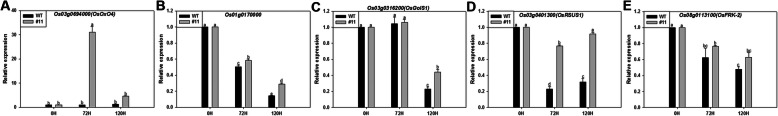
Expression of oxalate oxidase gene, raffinose/galactitol synthesis genes, fructose/sucrose synthetase genes in MoSDT1-transgenic line inoculated with rice blast strain at 0 h, 72 h, and 120 h. Relative expression levels of oxalate oxidase gene of *OsOXO4* (**a**), raffinose synthesis genes of *Os01g0170000* (**b**) and *OsGolS1* (**c**), fructose/sucrose synthetase genes of *OsRSUS1* (**d**), and *OsFRK-2*(**e**). Different letters (a, MoSDT1-transgenic rice line, differential metabolites three time points: b, 0 h; c, 72 h; d, 120 h) with a representative significant difference as *P* < 0.05

The level of genes, *OsLOX1/3* (Fig. 1a, b), *OsOPR1/7* (Fig. 1c, d), *OsJMT1* (Fig. 1e), and *OsHPL3* (Fig. 1f) related to jasmonic acid (JA) synthesis pathway in MoSDT1-transgenic rice detected by RT-qPCR showed significant up-regulation at 72 h. The genes involved in JA signaling pathway, such as *OsCOI1b* (Fig. 1g), *OsJAZ1* (Fig. 1h), *OsJAZ9* (Fig. 1i), and *OsMYC2* (Fig. 1j), as well as JA response genes, *JiOsPR10* (Fig. 1k) and *OsbHLH35* (Fig. 1l), were drastically inhibited at 72 h and 120 h. In addition, salicylic acid synthesis pathway gene *OsEDS1/OsPAD4* (Fig. 1m, n) and salicylic acid receptor gene *OsNPR4* (Fig. 1o) were significantly up-regulated at 72 h. The expression levels of raffinose synthase gene *Os01g0170000* (Fig. 2b), oxalate oxidase gene *OsOXO4* (Fig. 2a), and galactitol synthase gene *OsGolS1* (Fig. 2c) at 120 h were found to be lower at 0 h. Sucrose synthase gene *OsRSUS1* (Fig. 2d) and fructose synthase gene *OsFRK-2* (Fig. 2e) were down-regulated at 72 h and 120 h compared to 0 h, though their levels were significantly higher than infected wild-type rice. Cell-wall-associated kinases *OsCHI11* (Fig. S1A) and *OsWAK85* (Figure S1B) were significantly up-regulated in *M. oryzae*-infected MoSDT1-transgenic rice at 72 h, while the defense-related genes *OsPR10a* (Fig. S1C), *OsPR4a* (Fig. S1D), *OsPR5* (Fig. S1E) and *OsPR8* (Fig. S1F) were down-regulated. And the raw data of the above genes expression were shown in Supplementary file 2.

### Determination of major plant hormones in *Magnaporthe oryzae*-infected MoSDT1-transgenic rice

**Fig. 3:**
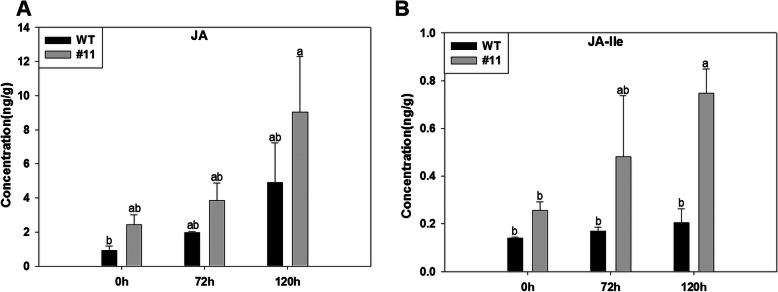
Plant hormone in MoSDT1-transgenic lines after *Magnaporthe oryzae* inoculation. Amount of JA (**a**), JA-Ile (**b**). Different letters indicate a, MoSDT1-transgenic rice line, differential metabolites three time points: b, 0 h; c, 72 h; d, 120 h, with a representative significant difference as *P* < 0.05

Multiple reaction monitoring (MRM) or selective reaction monitoring (SRM) is a considerably accurate and sensitive mass spectrometry platform that could specifically evaluate components among the complicated mixture. This technique was applied to quantify JA and JA-Ile content of *M. oryzae*-infected MoSDT1-transgenic rice strain #10 at three-time points (0 h, 72 h, and 120 h). The levels of JA (Fig. 3a; Supplementary file 2) and JA-Ile (Fig. 3b; Supplementary file 2) in MoSDT1-transgenic rice infected by *M. oryzae* 95234I–1b were significantly higher than the wild type rice. Although the content of JA and JA-Ile reached the highest level at 120 h, the amount of JA-Ile was much lower than the JA level (Fig. 3a and b; Supplementary file 2). This indicates that JA is the dominant form of jasmonic acid in infected transgenic rice.

### Analysis of the induced resistance of rice after treatment with exogenous compounds

**Fig. 4:**
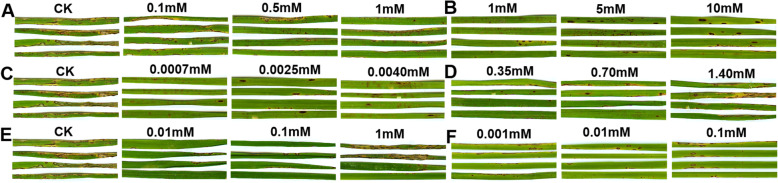
Symptom on leaves treated with metabolites inoculated with rice blast strain. Rice leaves were inoculated with rice blast fungus, 72 h later, three concentrations of exogenous galactol (**a**), tyramine (**b**), α-terpinene (**c**), L-glutamine (**d**), L-tryptophan (**e**) and dopamine hydrochloride (**f**) and sterilized water (CK) were applied onto the leaves

**Fig. 5:**
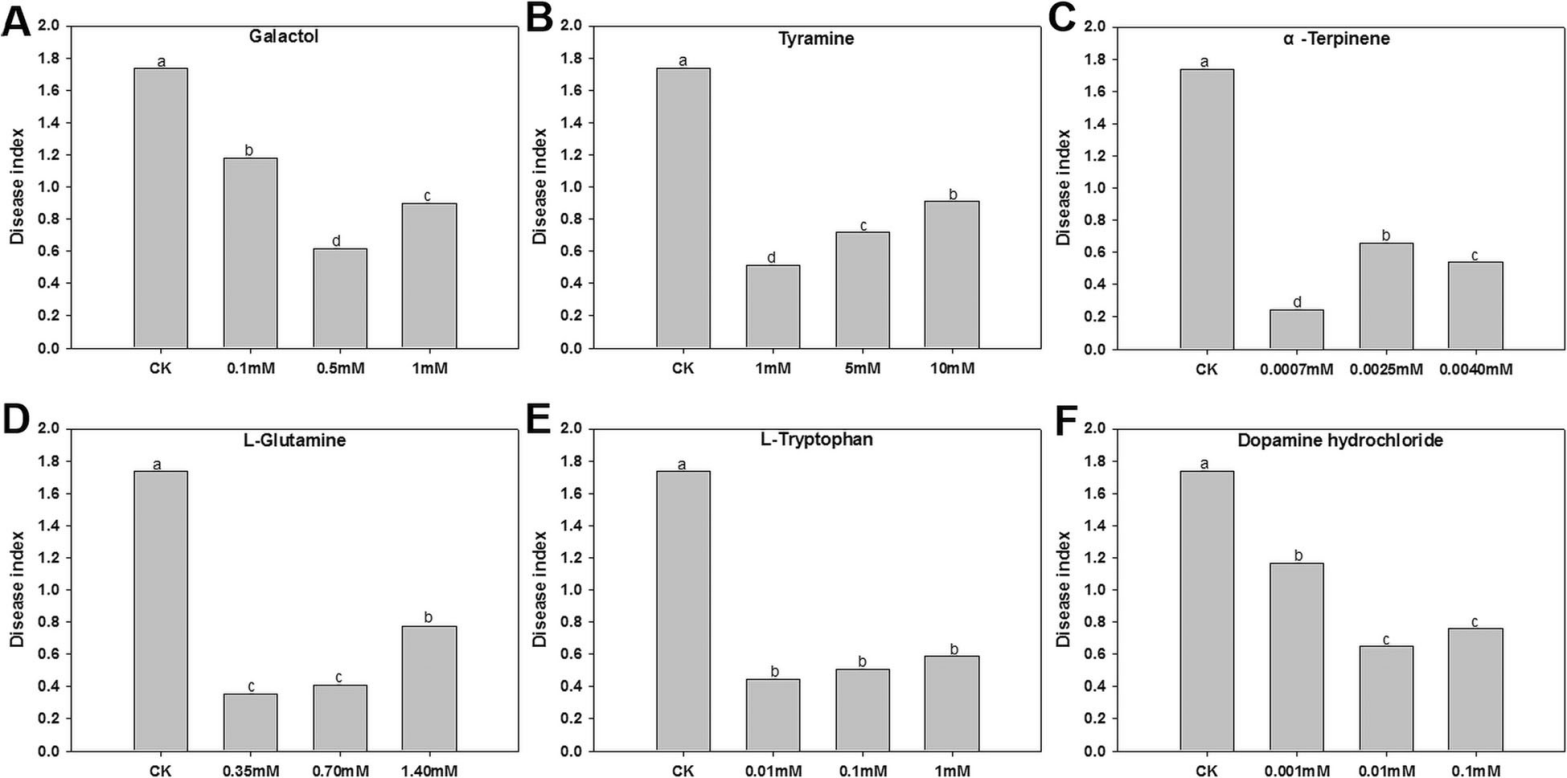
Disease index of leaves inoculated with rice blast strain and treated with exogenous metabolites. Exogenous treatment included three concentrations of six metabolites and sterilized water (CK). Different letters indicate a, MoSDT1-transgenic rice line, differential metabolites three time points: b, 0 h; c, 72 h; d, 120 h, with a representative significant difference as *P* < 0.05

Six metabolites of galactol, tyramine, L-glutamine, L-tryptophan, α-terpinene and dopamine were treated according to the three concentrations to the rice at 72 h after inoculation with rice blast fungus, and it was found that the symptoms of rice blast disease were drastically alleviated comparing to the control (Fig. 4). Six kinds of metabolites were exogenously treated with rice blast fungus 72 h after inoculation (the rice blast brown spots began to appear), index of the disease was dramatically dropped comparing to control (Fig. 5; Supplementary file 2). These six metabolites can be used as the best marker defense metabolites for exogenous application to improve rice blast resistance when brown spot type lesions of rice blast begin to appear.

## Discussion

### *M. oryzae*-infected MoSDT1-transgenic rice showed accumulation of multiple defensive metabolites and activation of multiple metabolic pathways

Previous studies revealed that when pathogens infect plants, the different metabolites induced by the infection play an essential role in strengthening the plant resistance and alleviate the disease symptoms. Our prior study confirmed that the heterologous expression of MoSDT1 in rice increases tiller numbers without affecting its nuclear morphology. Most importantly, it also resulted in a significant increase in resistance to *M. oryzae* in rice, mainly presented with early induction of massive callose deposition, ROS accumulation, cell death, and regulation of defense-related gene expression in infected MoSDT1-transgenic rice. Moreover, specific primary metabolites that participate in defense response accumulated in MoSDT1-transgenic rice [18]. To further investigate the biochemical mechanism of the enhanced resistance in *M. oryzae*-infected MoSDT1-transgenic rice, the metabolomics was analyzed to better explore the core metabolites and critical metabolic pathways of *M. oryzae*-infected MoSDT1-transgenic rice.

Organic oxygen compounds, organic acids, enzymes, and glycoside are the first category of metabolites produced by infected plants [2]. The accumulation of organic oxygen compounds such as sucrose, raffinose, galactinol, D-mannose, and maltotriose was identified at 0 h, 72 h, and 120 h in this study. D-mannose is mainly involved in synthesizing ascorbic acid, which is an antioxidant and cell reductant, and the improvement of antioxidant capacity is one of the characteristics of plant resistance [20]. Galactinol is also involved in inducing resistance in plant systems [21]. Glyceric acid is up-regulated in *Rhizoctonia solani*-infected resistant rice and participates in photorespiration and plant defense response regulation [9]. Thus, this indicates that the above illustrated organic oxygen compounds are unique metabolites accumulated in infected transgenic rice, and their biochemical and structural defense state with high antioxidant capacity were at the activation stage. The amino acid and carbohydrate metabolism pathways to which these accumulated metabolites belong were also activated. Therefore, the resistance in infected transgenic rice was strengthened.

Linoleic acid and linolenic acid are lipids that were found to accumulate at 72 h. Linolenic acid is a precursor of jasmonic acid [10] that plays a vital role in plant defense response. Thus up-regulated linolenic acid is a unique metabolite of infected transgenic rice. Subsequently, a further experiment validated an increase in jasmonic acid concentration at 120 h. Consistently, linoleic acid involved linoleic acid metabolism at 72 h.

L-glutamine is involved in stress response in plants [22]. Kan et al. found that glutamine can be used in the act of a signal molecule to modify expression patterns of plants. The prompt evocation of transcription factors indicates that glutamine can effectively intensify its signaling and engage with diverse signaling communication pathways to determine the growth of plant and its stress responses. Therefore, glutamine is an underlying effective amino acid that takes a critical role in nutrition and signaling transductions of plants [22]. L-tryptophan was found to accumulate at 72 h, indicating that the infected transgenic rice continues to grow. Oxalate has a dual role by inhibiting plant defense responses at the early stages of infection while also inducing reactive oxygen species and cell death at later stages of infection [23]. In our study, the concentration of oxalate decreased at 72 h, which was beneficial as it enhanced the ability of ROS clearance in infected transgenic rice and inhibited cell death. Most of these unique metabolites are involved in the citrate cycle (TCA cycle), thus indicating the activation of this pathway.

Benzenoids accumulated at different time-points in *M. oryzae*-infected transgenic rice, in which salicylic acid and anthranilic acid (Vitamin L1) accumulated at three-time points. Anthranilic acid (AA) is a vital precursor in the synthesis of major auxin, indole-3-acetic acid (IAA) [24], essential for maintaining the growth of infected transgenic rice. While salicylic acid plays a vital role in defense response [25], suggesting that MoSDT1 may play a role in balancing rice growth and defenses. Dopamine has an anti-aging effect that regulates the absorption and transport of nutrients by plants, which ultimately affects the overall growth of plants [26]. Tyramine accumulation, which is beneficial to increase resistance, was induced when barley was infected by *Bipolaris sorokiniana* [27]. Therefore, the accumulation of dopamine and tyramine in transgenic rice is beneficial to improve resistance. The metabolic pathways involved with tyrosine, tryptophan, and phenylalanine metabolism were thus activated.

Unclassified metabolites, such as vitexin, riboflavin, sinapyl alcohol, diosmetin, myo-inositol, D-biotin, and camptothecin, and alpha-pinene were found to accumulate at varying degrees at different time points. Both vitexin and apigenin have antiviral, antioxidant, and anti-cancer effects in plants [28]. L-tyrosine, an aromatic amino acid, is a precursor of lignin in plants of the grass family [29]. D-biotin plays an essential role in combating *M. oryzae* infection and promoting rice growth [30]. Meanwhile, D-biotin could also inhibit ROS accumulation and the appearance of lesion-mimic [30]. Sinapyl alcohols are products of lignin monomer synthesis [11]. Diosmetin, a flavonoid compound with antioxidant activity, protects plant tissues from oxidation [25]. Myo-inositol also plays a crucial role in the tolerance of abiotic stress [31]. These results indicate that these metabolites play an essential role in maintaining the growth of *Magnaporthe oryzae*-infected transgenic rice, keeping a dynamic balance of ROS accumulation and clearance, initiating structural defense response, and the restriction of spotted-lesion expansion and enhanced resistance of MoSDT1-transgenic rice.

Collectively, during the infection of MoSDT1-transgenic rice with *M. oryzae* at three-time points, organic oxygen compounds, lipids, and lipid-like molecules, benzenoids, organic acids, and derivatives, together with many unclassified primary defensive metabolites accumulated. Metabolic pathways involved with these metabolites are attributed to carbohydrate and amino acid metabolic pathways, which play a key role in stress response [19]. Therefore, these activated pathways participated in rice defense responses. This study found that at 72 h, the accumulation of defensive metabolites reached a peak, indicating that 72 h was the time for most active defense response, which was also the time point at which brown spotted lesions began to appear [32]. The increase in the number of accumulated defensive metabolites at this time is conducive to strengthen the defense response of MoSDT1-transgenic rice, restrict the spread of spotted lesions and increase the resistance of rice.

The accumulated metabolites identified in this study, such as diosmetin, D-biotin, tyramine, galactinol, alpha-Pinene, L-tryptophan, linolenic acid, sucrose, raffinose, fructose, and L-glutamine, can serve as core defensive metabolites for strengthening the resistance of rice. Hence, the predominantly enriched carbohydrate and amino acid metabolic pathways are the keys to the defense response.

### Crosstalk between multiple defensive metabolite response genes and plant hormones in *M. oryzae*-infected MoSDT1-transgenic rice

Linolenic acid and linoleic acid are precursors of jasmonic acid synthesis [10]. Salicylic acid and jasmonic acid are important hormones of plants, which take crucial roles in defense response and stable growth of rice [33]. Sun et al. examined changes in the phosphorylated proteomics of rice response to SA, suggesting that SA-mediated phosphorylation regulation may lead to different resistances of the two rice varieties [34]. *OsVQ13* positively regulates JA signal by activating OsMPK6-OsWRKY45 signaling pathway in rice [35]. JA reduces sensitivity of RBSDV infection by inhibiting the brassinosteroid (BR) pathway [36]. However, it is not clear about the JA/SA that contribute to defense response in rice.

In this study, these two metabolites and salicylic acid were identified to accumulate in *M. oryzae*-infected MoSDT1-transgenic rice at 72 h. Meanwhile, the contents of JA, JA-Ile, and salicylic acid were higher in infected transgenic rice than infected wild-type rice.

In this study, JA signaling pathway genes (*OsCOI1b*, *OsJAZ1*, *OsJAZ9,* and *OsMYC2)*, JA early response genes (*JiOsPR10* and *OsbHLH35*) and defense-related genes (*OsPR4a* and *OsPR10a)* were inhibited at 72 h and 120 h. Further analysis of JA synthesis pathway-related genes revealed that *OsLOX1/3*, *OsHPL3*, *OsOPR1*, *OsOPR7*, and *OsJMT1* were up-regulated at 72 h. In addition, *OsLOX3* accumulated linolenic acid at 72 h, and subsequently, the JA amount reached a peak at 120 h. It thus indicates that *OsLOX3* was mainly involved in converting linolenic acid to 13-hydroperoxide. *OsLOX1* and *OsLOX3* are similar in genetic structure and both are involved in pathogen defense responses [37], *OsHPL3* mediates SA and JA increase and enhances rice defense response [36, 38]. Increased JA and SA levels were noticed in this study, demonstrating that *OsHPL3* regulates JA and SA content in response to pathogen infection. In addition to its role in JA synthesis, *OsJMT1* is also primarily involved in responding to pathogen infection, during which *OsOPR7* got involved in JA biosynthesis in plants. However, *OsOPR1* plays a role in the rice self-defense response [39], indicating that the jasmonic acid synthetic pathway is activated, and genes in this pathway are involved in both jasmonic acid synthesis and defense response. It is, therefore, speculated that MoSDT1 might regulate jasmonic acid synthesis pathway-related genes to function in jasmonic acid synthesis and defense responses.

On the one hand, the up-regulation of *OsEDS1/OsPAD4* promotes SA biosynthesis, while on the other, it maintains SA-related resistant responses, strengthening the plant’s internal immune system [40]. It was reported that knock-out of *rrsRLK* gene can induce significant up-regulation of *OsPR1a*, *OsPR1b*, *OsLOX* and *RBBTI4* [41]. Thus indicating that the SA signaling pathway is activated, and the internal immune system is enhanced when transgenic rice is infected.

The expression level of the *OsOXO4* gene was significantly up-regulated in *M. oryzae*-infected MoSDT1-transgenic rice at 72 h. Meanwhile, the concentration of oxalate in infected transgenic rice was decreased, indicating that *OsOXO4* plays a role in scavenging oxalate at 72 h, avoiding the accumulation of reactive oxygen species and inhibition of cell death at the late stage of infection in transgenic rice. The expression level of raffinose synthase gene *Os01g0170000* and galactitol synthase gene *OsGolS1* in *M. oryzae*-infected MoSDT1-transgenic rice was higher than the wild type. Salicylic acid (SA) and nitric oxide (NO) also play a role in protecting plants from oxidative damage [42]; thus, the oxidative damage of infected transgenic rice is lower than the wild type rice. Therefore, *OsOXO4*, *Os01g0170000,* and *OsGolS1* play a role in protecting *M. oryzae*-infected MoSDT1-transgenic rice from infection with reactive oxygen species and inhibit drastic cell death at the late stage of the infection.

Chitinase and cell wall-associated kinases play a major role in plant defense and resistance to pathogenic fungal infection [43]. Cellulose synthase complex is regulated by metabolic signals related to plant carbon status [44]. Most of the fructose entering the cytoplasm, after phosphorylation by fructokinase (FRK), become components of cell wall synthesis functioning in xylem vessels, fibers, and overall vasculature development [45]. In this study, the expression levels of cell wall-associated kinase genes *OsCHI11* and *OsWAK85*, sucrose synthase gene *OsRSUS1* and fructose synthase gene *OsFRK–2* in MoSDT1-transgenic rice were significantly higher at late stages of the infection compared to the wild-type rice. Thus, indicating that these up-regulated genes are involved in cell wall remodeling or construction to enhance the physical barrier in MoSDT1-transgenic rice.

To summarize, SA, JA, raffinose, fructose, and sucrose accumulated in *M. oryzae-*infected MoSDT1-transgenic rice. Meanwhile, JA synthesis, SA synthesis/receptor gene, raffinose, fructose, and sucrose synthesis enzyme genes, and the cell wall-associated kinase gene were up-regulated. In contrast, genes involved in the JA signal gene were suppressed. These results indicate that JA and SA play a synergistic role in defense responses in MoSDT1-transgenic rice. ROS clearance-associated gene (*OsOXO4)* and raffinose synthesis genes (*Os01g0170000*, *OsGolS1)* play a role in protecting active oxygen damage. Moreover, the Fructose/sucrose synthase gene and cell wall-associated kinase genes are essential in cell wall remodeling.

### Improved resistance to *M. oryzae* by exogenous treatment with defensive metabolites

Studies have shown that biotin, galactitol, citrate, tyramine, raffinose, L-glutamine, L-tryptophan, dopamine, galactitol, terpinene, L-tryptophan and other metabolites serve as defensive signals in plant defense system [22, 46]. Rice infected with *M. oryzae* develop brown spotted lesions on leaves at 72 h and enter the time-point of dead body nutrition stage [32]. This is the time-point when metabolite accumulation is detected in infected transgenic rice, and most of these metabolites are associated with pathogen resistance. In addition, studies have also shown that exogenous application of metabolites (such as GABA, dopamine, proline, tyramine) can increase plant resistance [26, 47–49]. From the metabolites accumulated in MoSDT1-transgenic rice infected by *M. oryzae*, six metabolites (galactinol, tyramine, L-glutamine, L-tryptophan, pinene and dopamine) were selected to treat *M. oryzae*-infected rice in two ways exogenously. Among these six metabolites, the treatment significantly increased rice resistance at 72 h, and its low concentration also played a significant role in improving rice resistance. Therefore, low-concentration metabolites as defensive weapons have the most apparent control response when brown spotted lesions of rice blast begin to form (72 h); however, the underlying mechanism involved requires further investigation.

To summarize, among the defensive metabolites accumulated at different stages of the pathogen-infected plant, six defensive metabolites were selected to treat the plants exogenously at a specific time-point of the infection to improve plant resistance. Thus this makes exogenous application of defensive metabolites an environmental control strategy for future applications.

## Conclusions

This study provided an insight into the metabolome alterations in *MoSDT1*-transgenic rice infected by *M.oryzae*. The result shows that more primary defensive metabolites accumulated in *MoSDT1*-transgenic rice at the late stage of infection, moreover, some defensive metabolites could exogenously alleviate blast symptoms of infected rice, indicating that they could be utilized as an optimal defense markers during *M.oryzae* infection. The synergistical interactions of SA-JA regulate expressions of defense-related genes to enhance defensive responses in rice. Taken together, our results disclosed the metabolic mechanism of *MoSDT1*-transgenic rice in defending against *M.oryzae* infection, providing valuable evidence for strengthening rice the broad-spectrum resistance of rice and blast disease management.

## Methods

### Rice growth and rice blast strain culture

MoSDT1-transgenic line #10, WT variety (*O. sativa* ssp. *japonica* cv. Ilmibyeo), and Ilmibyeo is a Korean domestic rice cultivars, representing white rice in Korean market, was developed from the three-way cross of Milyang `96//Milyang `95/Seomjinbyeo [50], and rice blast strain 95234I-1b were maintained in the laboratory as previously described [18]. The method for cultivating rice seedling was also based on the report of Wang et al. [18]. Rice blast strain 95234I-1b was previously isolated in my lab. In brief, 95234I-1b strain exhibited with strong virulence and could infect rice variety ilimi which further result with severe symptoms of rice blast disease. However, overexpression of *MoSDT1* in ilimi significantly increased the resistance of rice to this strain [18]. In order to further study the metabolic mechanism of interactions between MoSDT1-transgenic rice and 95234I-1b, we proceed by using 95234I-1b to infect MoSDT1-transgenic rice as research materials.

Rice seeds of #10 and WT were sterilized in 75% alcohol for 1 min, washed three times in sterile water, followed by sterilizing in 1.5% hypochlorite solution for 5 min, and finally washed three times in sterile water. The seeds were then germinated in 5% hygromycin aqueous solution and sterile water, respectively at 28 °C. The seeds were sown in plastic buckets and plates and each treatment was repeated three times. The seedlings in plastic buckets were cultivated for 14 days according to conventional management in the greenhouse.

The rice blast strain of 95,234 I–1b was cultured in PSA medium (200 g potatoes, 10 g sugar, 15 g agar powder, 1 L sterile water) at 28 °C. After the mycelium grows all over the Petri dish, the surface mycelium was scraped away, and the spores were washed using sterilized water to prepare spore suspension. The concentration of spore suspension was adjusted to 1× 10^5^/mL for spray inoculation. The spore suspension was inoculated on 14-day-old rice leaves and the inoculated leaves were incubated at 28 °C for 24 h and moved to a greenhouse for growth. Samples were collected at 0 h, 72 h, and 120 h after inoculation, and six biological replicates were used for UHPLC-Q-TOF MS analysis. The leaves of six individual plants were sampled for each replication. Three biological replicates were used for qRT-PCR analysis and leaves of three individual plants were sampled for each replicate.

### UHPLC-Q-TOF MS analysis

The AB Triple TOF 5600/6600 mass spectrometer (AB Sciex, USA) and Q-TOF MS/MS were combined for analysis. Rice samples were ground in liquid nitrogen and added with 1 mL methanol:acetonitrile:aqueous solution (2:2:1, v:v:v), vortexed for 60 s and underwent ultrasound at low temperature for two times (30 min for each time). It was then placed at − 20 °C for 1 h, centrifuged at 14000 rcf at 4 °C for 20 min, frozen-dried and preserved at − 80 °C. The samples were separated by Agilent 1290 Infinity LC UHPLC column (Agilent, USA). The column was set at 25 °C and the flow rate was set at 0.3 mL/min. The composition of mobile phase was A: water + 25 mM ammonium acetate + 25 mM ammonia water, B: ethoxine. Gradient elution procedure was as follows: 0–5 min, 95% B; 0.5–7 min, B changed linearly from 95 to 65%; 7–8 min, B changed linearly from 65 to 40%, B maintained at 40% during 8–9 min. The linear change of B from 40 to 95% was for 9–9.1 min. B stayed at 95% for 9–12 min. The samples were placed in 4 °C auto-sampler during the whole process. MS detection was performed using electrospray (ESI) positive and negative ion modes. The samples were separated by UHPLC and analyzed by Agilent6550 mass spectrometer. The ESI source conditions were as follows: Gas Tem: 250 °C, Drying Gas: 16 L/min, Nebulizer: 20 psig, Sheath Gas Temp: 400 °C, Sheath Gas Flow: 12 l/min, Vcap: 3000 v, Nozzle voltage: 0 v, fragments: 175 v, Mass Range: 50–1200, the Acquisition rate: 4 Hz, cycle time: 250 ms. After the samples were tested, AB Triple TOF 6600 mass spectrometer identified the metabolites and collected the first and second level spectra. The ESI source conditions were as follows: Ion Source Gas1 (Gas1):40, Ion Source Gas2 (Gas2):80, Curtain Gas (CUR) 30, Source temperature: 650 °C, IonSapary Voltage Floating (ISVF) ±5000 V (plus or minus two modes), and secondary mass spectrometry obtained through information-dependent acquisition (IDA) and high sensitivity mode, Declustering potential (DP): ±60 V (plus or minus two modes), Collision Energy: 35 ±eV, IDA: Exclude isotopes within 4 Da, candidate ions to monitor per cycle: 10. Data collection was segmented according to the mass range, 50–300, 290–600, 590–900, and 890–1200, so as to expand the collection rate of the second-level atlas. The data generated from each method were collected four times in each section. The original data were been transformed into. MzXML format by Proteo Wizard, and then XCMS procedure was used for peak alignment, retention time correction, and extraction of peak area. The accurate mass number matching (< 25 ppm) and secondary map matching were used to search the database for metabolite structure identification. After the data were pretreated with pareto-scaling, multidimensional statistical analysis was performed, including unsupervised principal component (PCA) analysis, supervised partial least squares discriminant analysis (PLS-DA), and orthogonal partial least squares discriminant analysis (OPLS-DA). Unidimensional statistical analysis included Student’s t-test and variable-multiple analysis.

### Expression analysis of defense-related genes in rice

RT-qPCR was performed with MoSDT1-transgenic line and WT inoculated with rice blast strain at 0 h, 72 h, and 120 h as previously described by Wang et al. [18]. cDNA reverse transcription was performed by Bio-Rad CFX96 TM Real-Time System (Bio-Rad, USA). The operation was carried out in accordance with the GoScript TM Reverse Transcription System A5001 kit (TransGen Biotechnology Co., Ltd., Beijing). qRT-PCR fluorescent dye, SYBR Premix Ex Taq II, was prepared for the reaction system according to the mentioned instructions with a total volume of 20 μL. Primer 5 was used to design primers for defense-related genes (sequences of primers are shown in Supplementary Table 6). Bio-Rad CFX96 TM real-time System (Bio-Rad, USA) was used for product amplification and fluorescence signal detection.

### Determination of plant endogenous hormone assay

Plant endogenous hormone assays were performed in accordance with the method of Benton et al. [51]. Rice leaves (1 g) were ground using liquid nitrogen, 80 mg was taken in a 2 mL centrifuge tube and added with 50 μL of the internal standard solution along with 1 mL acetonitrile aqueous solution (1% FA). It was shaken and mixed well for 2 min, kept at 4 °C for 12 h in the dark, centrifuged at 14000 ×g for 10 min, 800 μL of the supernatant was taken and dried with liquid nitrogen. It was reconstituted with 100 μL of acetonitrile-water (1:1, v/v), centrifuged at 14000 ×g for 10 min, and the supernatant was used for analysis. The samples were separated by Agilent 1290 Infinity LC Ultra-high Performance Liquid Chromatography platform (Mobile phase: Liquid A was 0.05% aqueous FA solution, and liquid B was 0.05% FA acetonitrile). The sample was placed in a 4 °C auto-sampler set at 45 °C, at a flow rate of 400 μL/min, and the loading volume was 2 μL. Correspondingly liquid phase gradient was as follows: 0–10 min, B liquid changes from 2 to 98% linearly; 10–11.1 min, B liquid changes from 98 to 2% linearly; 11.1–13 min, B liquid is maintained at 2%. A QC sample was placed for a certain number of experimental samples in a sample order to detect and evaluate the stability and repeatability of the system. Mass spectrometry analysis was performed in positive/negative ion mode by using a 5500 QTRAP mass spectrometer (AB SCIEX). The 5500 QTRAP ESI conditions were as follows: source temperature 500 °C, ion Source Gas1 (Gas1): 45, Ion Source Gas2 (Gas2): 45, Curtain gas (CUR): 30, ionSapary Voltage Floating (ISVF)–4500 V; MRM mode was used to detect the pair of ions. The peak area and retention time were calculated using Multiquant software. The amount of phytohormone measured in the sample was calculated based on the standard curve.

### Evaluation of exogenous compound treatment on rice resistance

Firstly, rice leaves were inoculated with spores of *M. oryzae* strain 95234I–1b, and then sprayed with exogenous compounds after 72 h at three concentrations (Supplementary Table 7), and the concentrations of six compounds were prepared [21, 24, 26]. Disease index =∑ (number of leaves at each grade × representative values ​​at all grades)/(review total leaf number × highest grade representative value)× 100 [18]. All experiments were carried out in the lab/under greenhouse. Conditions were controlled at 28 °C/26 °C (day/night) with 16 h of light and 8 h of dark and a relative humidity of 50%. The experiments were performed with biological repeats and technical repeats three times each.

## Supplementary Information

**Additional file 1: Figure S1.** Expression of cell wall-associated kinases and defense-related genes in MoSDT1-transgenic line inoculated with rice blast strain at 0 h, 72 h, and 120 h. Cell wall-associated kinase genes include *OsCHI11* and *OsWAK85* (Figure S1 A, B). Defense-related genes include *OsPR10a* (Figure S1 C), *OsPR4a* (Figure S1 D), *OsPR5* (Figure S1 E), and *OsPR8* (Figure S1 F). Different letters indicate a, MoSDT1-transgenic rice line, differential metabolites three time points: b, 0 h; c, 72 h; d, 120 h with a representative significant difference as *P* < 0.05.

**Additional file 2: Table S1.**

**Additional file 3: Table S2.** Amino acid metabolic pathway enrichment analysis of differential metabolites between *MoSDT1*-transgenic line challenged with blast strain.

**Additional file 4: Table S3.** Carbohydrate metabolic pathway enrichment analysis of differential metabolites between MoSDT1 transgenic line challenged with blast strain.

**Additional file 5: Table S4.** Nitrogen metabolic pathway and ABC transporters enrichment analysis of differential metabolites between MoSDT1 transgenic line challenged.

**Additional file 6: Table S5.** Partial differential metabolites involved in main KEGG pathway between *MoSDT1*-transgenic line challenged without and with blast strain.

**Additional file 7: Table S6.** Primers used for qRT-PCR.

**Additional file 8: Table S7** Different concentrations of six compounds.

**Additional file 9.**

**Additional file 10.**

## Data Availability

All data generated or analysed during this study are included in this article [and its supplementary information files]. The datasets used and/or analysed during the current study are available from the corresponding author on reasonable request.

## Abbreviations

MoSDT1: *Magnaporthe oryzae* Systemic Defense Trigger 1
UHPLC/Q-TOF-MS: Ultra-high performance liquid chromatography-quadrupole time-of-flight mass spectrometry
SA: Salicylic acid
JA: Jasmonic acid
PAMP/MAMP: Pathogen-associated molecular patterns/microbe-associated molecular pattern
PTI: PAMP/MAMP-triggered immunity
ETI: Effector-triggered immunity
ABC transporters: ATP-binding cassette transporter
qRT-PCR: Quantitative Real-time Polymerase Chain Reaction
KEGG: Kyoto Encyclopedia of Genes and Genome
OsLOX1/3: Rice lipoxygenase1/3
OsOPR1/7: Rice oxophytodienoate reductase1/7
OsJMT1: Rice jasmonate O-methyltransferase
OsHPL3: Rice hydroperoxidelyase 3
OsCOI1b: Rice coronatine insensitive 1b
OsJAZ1/9: Rice jasmonate ZIM-domain protein 1/9
OsEDS1/OsPAD4: Rice enhanced disease susceptibility 1/ rice phytoalexin Deficient 4
JiOsPR10: Jasmonate inducible pathogenesis-related class 10 protein
OsbHLH35: Rice basic helix-loop-helix 35
OsNPR4: Rice receptor gene 4
OsOXO4: Rice oxalate oxidase 4
OsGolS1: Rice galactinol synthase 1
OsFRK–2: Rice Fructose synthase − 2
OsRSUS1: Rice sucrose synthase 1
OsCHI11: Rice chitinase 11
OsPR8: Rice pathogenicity-related gene 8
OsPR4a: Rice pathogenicity-related gene 4a
OsPR10a: Rice pathogenicity-related gene 10a
OsPR5: Rice pathogenicity-related gene 5
OsWAK85: Rice wall associated kinases 85
