# Supplementary figures and images for: Magnaporthe oryzae systemic defense trigger 1 (MoSDT1)-mediated metabolites regulate defense response in Rice
Source: BMC Plant Biol. 2021 Jan 11;21:40. doi: 10.1186/s12870-020-02821-6 (PMC7802159; doi:10.1186/s12870-020-02821-6)

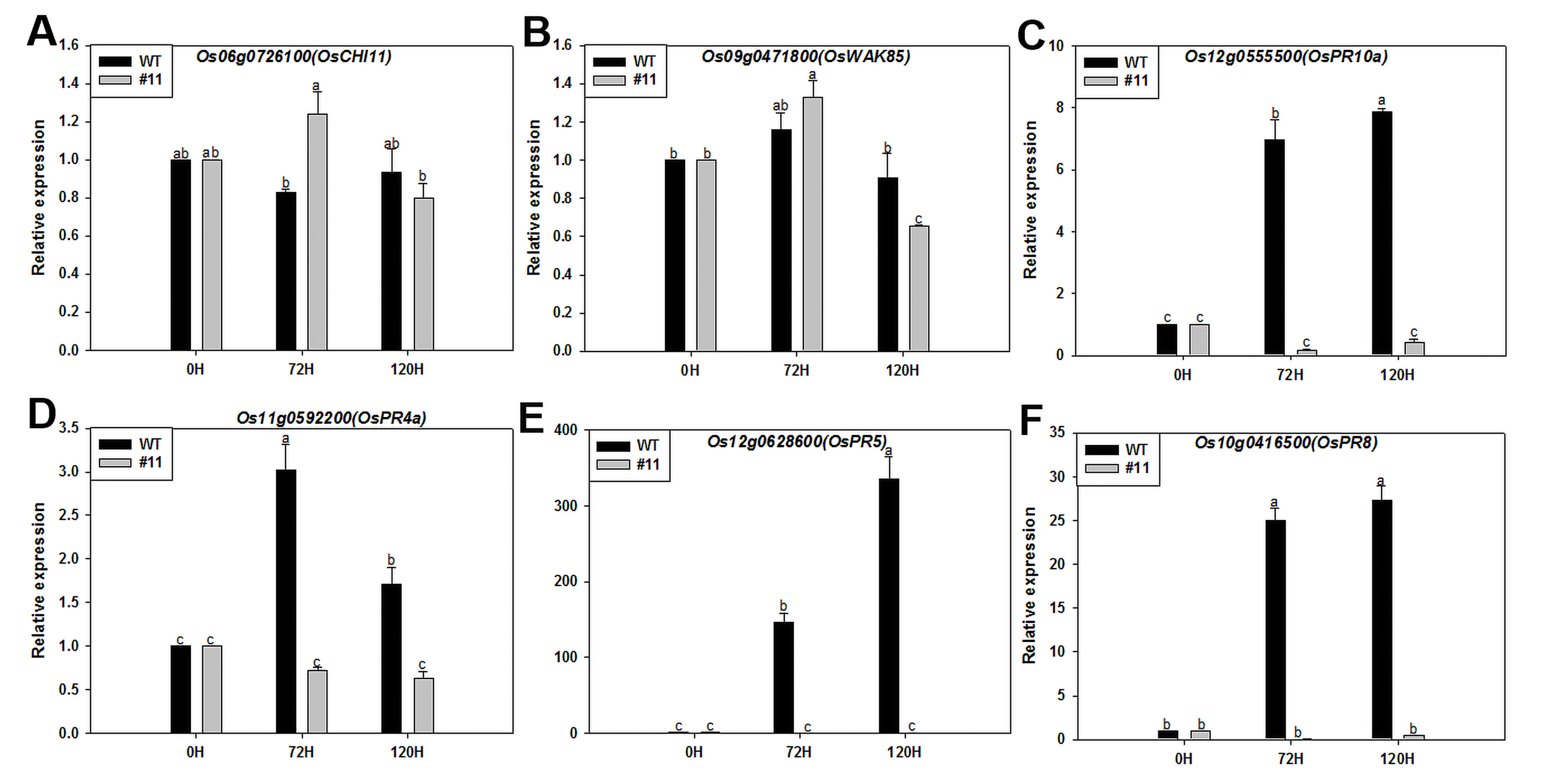

Supplement: Supplementary file 1 — Additional file 1: Figure S1. Expression of cell wall-associated kinases and defense-related genes in MoSDT1-transgenic line inoculated with rice blast strain at 0 h, 72 h, and 120 h. Cell wall-associated kinase genes include OsCHI11 and OsWAK85 (Figure S1 A, B). Defense-related genes include OsPR10a (Figure S1 C), OsPR4a (Figure S1 D), OsPR5 (Figure S1 E), and OsPR8 (Figure S1 F). Different letters indicate a, MoSDT1-transgenic rice line, differential metabolites three time points: b, 0 h; c, 72 h; d, 120 h with a representative significant difference as P < 0.05. [file 12870_2020_2821_MOESM1_ESM.tif]
