# Supplementary material for: Magnaporthe oryzae systemic defense trigger 1 (MoSDT1)-mediated metabolites regulate defense response in Rice
Source: BMC Plant Biol. 2021 Jan 11;21:40. doi: 10.1186/s12870-020-02821-6 (PMC7802159; doi:10.1186/s12870-020-02821-6)
Supplement: Supplementary file 3 — Additional file 3: Table S2. Amino acid metabolic pathway enrichment analysis of differential metabolites between MoSDT1-transgenic line challenged with blast strain. [file 12870_2020_2821_MOESM3_ESM.docx]

**Table S2 Amino acid metabolic pathway enrichment analysis of differential metabolites between *MoSDT1*-transgenic line challenged with blast strain**

| Mo11 vs WT (0h)^b^ | | Mo11 vs WT (72h)^c^ | | Mo11 vs WT (120h)^d^ | |
| --- | --- | --- | --- | --- | --- |
| Pathways | p value | Pathways | p value | Pathways | p value |
| beta-Alanine metabolism | 0.0002 | Alanine, asparate and glutamate metabolism | 0.0039 | Alanine, asparate and glutamate metabolism | 0.0028 |
| Alanine, asparate and glutamate metabolism | 0.0011 | beta-Alanine metabolism | 0.0063 | Glutathione metabolism | 0.048 |
| Phenylalanine, tyrosine and tryptophan biosynthesis | 0.018 | Arginine biosynthesis | 0.016 |  |  |
| Tyrosine metabolism | 0.025 | Phenylalanine, tyrosine and tryptophan biosynthesis | 0.049 |  |  |
| Arginine biosynthesis | 0.028 | Glutathione metabolism | 0.012 |  |  |
| Butanoate metabolism | 0.033 |  |  |  |  |
| Glutathione metabolism | 0.023 |  |  |  |  |

Differential metabolites in *MoSDT1*-transgenic rice line (a), differential metabolites at 0h (b), 72h (c), 120h (d) in *MoSDT1*-transgenic rice line inoculated with rice blast strain.
