# Supplementary material for: Magnaporthe oryzae systemic defense trigger 1 (MoSDT1)-mediated metabolites regulate defense response in Rice
Source: BMC Plant Biol. 2021 Jan 11;21:40. doi: 10.1186/s12870-020-02821-6 (PMC7802159; doi:10.1186/s12870-020-02821-6)
Supplement: Supplementary file 4 — Additional file 4: Table S3. Carbohydrate metabolic pathway enrichment analysis of differential metabolites between MoSDT1 transgenic line challenged with blast strain. [file 12870_2020_2821_MOESM4_ESM.docx]

**Table S3 Carbohydrate metabolic pathway enrichment analysis of differential metabolites between MoSDT1 transgenic line challenged with blast strain**

| Mo11 vs WT (0h)^b^ | | Mo11 vs WT (72h)^c^ | | Mo11 vs WT (120h)^d^ | |
| --- | --- | --- | --- | --- | --- |
| Pathways | p value | Pathways | p value | Pathways | p value |
| Galactose metabolism | 4.81E-07 | Galactose metabolism | 6.43E-08 | Galactose metabolism | 4.44E-06 |
| Glyoxylate and dicarboxylate metabolism | 0.0017 | Glyoxylate and dicarboxylate metabolism | 7.4E-05 | Glyoxylate and dicarboxylate metabolism | 3.86E-05 |
| Citrate cycle(TCA) | 0.0023 | Citrate cycle(TCA) | 0.0011 | Citrate cycle(TCA) | 4.99E-05 |
| Amion sugar and nucleotide sugar metabolism(8) | 0.012 | Aminoacyl t-RNA biosynthesis | 0.033 | Biosynthesis of unsaturated fatty acids | 0.00095 |
| Aminoacyl t-RNA biosynthesis | 0.017 | Fructose and mannose metabolism | 0.038 | Fatty acid biosynthesis | 0.0041 |
| Glycerolipid metabolism | 0.018 | Linoleic acid metabolism | 0.027 | Linoleic acid metabolism | 0.022 |
| Starch and sucrose metabolism | 0.021 | Pentose phosphate pathway | 0.049 | Oxidative phosphorylation | 0.046 |
| Pantothenate and CoA biosynthesis | 0.046 |  |  |  |  |
| Ascorbate and aldarate metabolism | 0.047 |  |  |  |  |
| Linoleic acid metabolism | 0.046 |  |  |  |  |

Differential metabolites in MoSDT1 transgenic rice line (a), differential metabolites at 0h (b), 72h (c), 120h (d) in MoSDT1 transgenic rice line inoculated with rice blast strain.
