# Supplementary material for: Magnaporthe oryzae systemic defense trigger 1 (MoSDT1)-mediated metabolites regulate defense response in Rice
Source: BMC Plant Biol. 2021 Jan 11;21:40. doi: 10.1186/s12870-020-02821-6 (PMC7802159; doi:10.1186/s12870-020-02821-6)
Supplement: Supplementary file 5 — Additional file 5: Table S4. Nitrogen metabolic pathway and ABC transporters enrichment analysis of differential metabolites between MoSDT1 transgenic line challenged. [file 12870_2020_2821_MOESM5_ESM.docx]

**Table S4 Nitrogen metabolic pathway and ABC transporters enrichment analysis of differential metabolites between MoSDT1 transgenic line challenged**

**with blast strain**

| Mo11 vs WT (0h)^b^ | | Mo11 vs WT (72h)^c^ | | Mo11 vs WT (120h)^d^ | |
| --- | --- | --- | --- | --- | --- |
| Pathways | p value | Pathways | p value | Pathways | p value |
| Purine metabolism | 1.66E-06 | Pyrimidine metabolism | 2.46E-07 | Pyrimidine metabolism | 0.013 |
| Pyrimidine metabolism | 2.11E-06 | Purine metabolism | 0.00026 | Nicotinate and nicotiamide metabolism | 0.030 |
| Zeatin metabolism  Nicotinate and nicotiamide metabolism | 0.026  0.021 |  |  |  |  |
|  |  |  |  |  |  |
| ABC transporters | 6.59E-05 | ABC transporters | 3.51E-05 | ABC transporters | 7.4E-05 |

Differential metabolites in MoSDT1 transgenic rice line (a), differential metabolites at 0h (b), 72h (c), 120h (d) in MoSDT1 transgenic rice line inoculated with rice blast strain.
