# Supplementary material for: Magnaporthe oryzae systemic defense trigger 1 (MoSDT1)-mediated metabolites regulate defense response in Rice
Source: BMC Plant Biol. 2021 Jan 11;21:40. doi: 10.1186/s12870-020-02821-6 (PMC7802159; doi:10.1186/s12870-020-02821-6)
Supplement: Supplementary file 6 — Additional file 6: Table S5. Partial differential metabolites involved in main KEGG pathway between MoSDT1-transgenic line challenged without and with blast strain. [file 12870_2020_2821_MOESM6_ESM.docx]

**Table S5 Partial differential metabolites involved in main KEGG pathway between *MoSDT1*-transgenic line challenged without and with blast strain**

| Metabolites | Pathway | Mo11 vs WT (0h)^b^ | | Mo11 vs WT (72h)^c^ | | Mo11 vs WT (120h)^d^ | |
| --- | --- | --- | --- | --- | --- | --- | --- |
|  |  | Fold change | p value | Fold change | p value | Fold change | p value |
| Galactinol | Galactose metabolism | 1.675 | 0.00018 | 1.575 | 0.059 | 2.949 | 1.43E-07 |
| D-Mannose | Fructose and mannose metabolism | 1.644 | 0.051 | 1.607 | 0.044 | 1.605 | 0.0258 |
| D-Biotin | Biotin metabolism/ | 2.548 | 0.00019 | 2.314 | 0.048 | 1.651 | 2.29-05 |
| Salicylic acid | Phenylalanine metabolism | 1.823 | 0.0705 | 1.786 | 9.61E-05 | 1.442 | 3.93E-05 |
| Shikimate | Phenylalanine, tyrosine and tryptophan biosynthesis | 1.435 | 0.000792 | 1.841 | 0.0041 | 1.533 | 4.85E-06 |
| 16-Hydroxypalmitic acid | **-** | 2.419 | 6.46E-05 | 1.196 | 0.029 | 1.121 | 0.097 |
| Pregnenolone | **-** | 3.556 | 2.02E-07 | 2.084 | 1.78E-05 | 2.853 | 1.08E-07 |
| Matairesinol | **-** | 2.128 | 0.067 | 1.491 | 0.038 | 1.498 | 0.0054 |
| Camptothecin | **-** | 3.749 | 0.0031 | 1.766 | 0.033 | 2.153 | 5.83E-06 |
| Glyceric acid | Pentose phosphate pathway | **-** | **-** | 1.522 | 0.010 | 1.196 | 0.049 |
| Sucrose | Galactose metabolism | 0.3111 | 0.00054 | 1.536 | 0.0015 | 1.607 | 0.00015 |
| Raffinose | Galactose metabolism | **-** | **-** | 1.190 | 0.087 | 1.849 | 2.48E-06 |
| L-Glutamine | Arginine biosynthesis | 2.798 | 4.66E-06 | 2.659 | 1.20E-07 | 3.251 | 1.52E-08 |
| Maltotriose | ABC transporters | **-** | **-** | 1.586 | 0.0094 | 2.061 | 2.24E-05 |
| Sinapyl alcohol | **-** | 0.7791 | 0.0036 | 0.589 | 3.53E-07 | 0.601 | 4.17E-06 |
| D-Tagatose | Galactose metabolism | 1.546 | 0.0037 | 0.443 | 0.00065 | 1.336 | 0.0070 |
| Anthranilic acid | Tryptophan metabolism | 1.498 | 1.82E-05 | 1.251 | 0.0081 | 1.219 | 0.00024 |
| Dopamine | Tyrosine metabolism | 1.300 | 0.045 | 1.451 | 0.00030 | **-** | **-** |
| Xylitol | ABC transporters | 1.384 | 0.0672 | - | - | 2.664 | 1.96E-07 |
| Trehalose | Starch and sucrose metabolism | 3.492 | 0.034 | 0.499 | 0.0095 | 1.456 | 0.00042 |
| Pantothenate | beta-Alanine metabolism | 1.343 | 6.11E-06 | 1.369 | 0.00063 | - | **-** |
| alpha-Pinene | - | **-** | **-** | **2.891** | **0.00430** | **-** | **-** |
| Linoleic acid | Biosynthesis of unsaturated fatty acids | 0.747 | 0.0050 | 1.397 | 0.054 | 0.865 | 0.096 |
| myo-Inositol | Galactose metabolism | 0.934 | 0.086 | 1.656 | 0.066 | - | - |

Differential metabolites in *MoSDT1*-transgenic rice line (a), differential metabolites at 0h (b), 72h (c), 120h (d) in *MoSDT1*-transgenic rice line inoculated with rice blast strain.
