# Supplementary material for: Magnaporthe oryzae systemic defense trigger 1 (MoSDT1)-mediated metabolites regulate defense response in Rice
Source: BMC Plant Biol. 2021 Jan 11;21:40. doi: 10.1186/s12870-020-02821-6 (PMC7802159; doi:10.1186/s12870-020-02821-6)
Supplement: Supplementary file 7 — Additional file 7: Table S6. Primers used for qRT-PCR. [file 12870_2020_2821_MOESM7_ESM.docx]

**Table S6. Primers used for qRT-PCR.**

| Gene name | Accession number | Forward primer(5'-3') | Reverse primer(5'-3') |
| --- | --- | --- | --- |
| OsLOX1 | Os02g0194700 | GTCCTGCTTCATCGTCCTGTG | ACCCAAGTGAGCAGCACAACA |
| OsLOX3 | Os03g0699700 | CCGACCAAAACAATGAGGGC | GCACACCATGCTTGTCCATC |
| OsOPR1 | Os06g0216300 | AGCACAATGACCTTTGATTCTTC | TTCCTGCAGATATGGCACGTC |
| OsOPR7 | Os08g0459600 | TTGGGTTGGAAGCAGTGGAG | GGATCGGGCGTGTAGAATGT |
| OsJMT1 | Os06g0314600 | ATCGATGAGCTTCCGTGCG | GCATAAGCCTGTGATGGCCT |
| OsHPL3 | Os02g0110200 | TAGTGTCGGCACCATCCAAC | TCACTTCCTTGCCATGTTGT |
| OsCOI1b | Os05g0449500 | GTAATGTTGGGGAAACAGATG | AAGCTTGCTCACTGAAGCAACAA |
| OsJAZ1 | Os03g0181100 | CAGCAGGTTGGTGAGCAAAG | TCCATCCCTGATGCTTCCAT |
| OsJAZ9 | Os03g0180800 | CGGTCGAGTTGGAAGATGGTT | GGTCAGGCTCGGCGAAAT |
| OsMYC2 | Os10g0575000 | CTAGCGAGGAAACCCAATCG | CCATCCATCCATCCTAACAC |
| JiOsPR10 | Os03g0300400 | CAACCCGGACGCTTACAACT | CCATTCTCCGACAGCAACCA |
| OsbHLH35 | Os04g0301500 | GTCATTTTTGCGACACCCCA | CCTCGCAAGAAAACAACCACC |
| OsEDS1 | Os09g0392100 | CATTCCAAGAACGAGGACACTG | CAAGACTCAAGGCTAGAACCGA |
| OsPAD4 | Os11g0195500 | CCAACATGTACCGCATCAAG | GGTTGTTTCGGTGGTAGTGG |
| OsNPR4 | Os01g0837000 | CAACGTCGAGCAAATGTACG | TCAAGCACTGGAGTCAGCTC |
| OsOXO4 | Os03g0694000 | CGACACCGGGAACAGGTACT | GTCTTGCCAACGTTGAACT |
| Raffinose synthase | Os01g0170000 | CCAGGATGCGGAGTTCA | CTAGTAAAAGTACTCGACA |
| OsGolS1 | Os03g0316200 | GCGGCGTACCTCTGCATC | AAGCCTCGTTACATTTGTG |
| OsRSUS1 | Os03g0401300 | CCTCGCACCAATCCATTCCA | CCTCTTGGCTCCTCAAGCCTTC |
| OsFRK-2 | Os08g0113100 | GGGATGCAGGTACTTCACCAA | TCCTCGTTGTGGAAGATGGAG |
| OsCHI11 | Os06g0726100 | GTTCTGGTTCTGGATGACGC | GCCGTTGATGATGTTGGTGA |
| OsWAK85 | Os09g0471800 | GCTCACCTACTACCACAGGC | AGGGGATATGCACCAACACG |
| OsPR10a | Os12g0555500 | TGTGTGGCCAAGCTCAAGGT | GACGAGGTAGTCCTCGATCA |
| OsPR4a | Os11g0592200 | GATGCCAACAAACCGTTGTC | CGCAATTATTGTCGCACCTG |
| EF-1α | Os03g0177400 | CAAGCTGAAGGGTATTGACCA | TTGTCAAGAACCCAGGCATAC |
