# Supplementary material for: Magnaporthe oryzae systemic defense trigger 1 (MoSDT1)-mediated metabolites regulate defense response in Rice
Source: BMC Plant Biol. 2021 Jan 11;21:40. doi: 10.1186/s12870-020-02821-6 (PMC7802159; doi:10.1186/s12870-020-02821-6)
Supplement: Supplementary file 8 — Additional file 8: Table S7 Different concentrations of six compounds. [file 12870_2020_2821_MOESM8_ESM.docx]

**Table S7 Different concentrations of six compounds**

| Compounds | A | B | C | D | E | F |
| --- | --- | --- | --- | --- | --- | --- |
|  | Galactol | Tyramine | α-Terpinene | L-Glutamine | L-Tryptophan | Dopamine hydrochloride |
| Concentration | 0.1 | 1 | 0.0007 | 0.35 | 0.01 | 0.001 |
| (mM) | 0.5 | 5 | 0.0025 | 0.70 | 0.1 | 0.01 |
|  | 1 | 10 | 0.0040 | 1.40 | 1 | 0.1 |
