# Supplementary material for: Magnaporthe oryzae systemic defense trigger 1 (MoSDT1)-mediated metabolites regulate defense response in Rice
Source: BMC Plant Biol. 2021 Jan 11;21:40. doi: 10.1186/s12870-020-02821-6 (PMC7802159; doi:10.1186/s12870-020-02821-6)
Supplement: Supplementary file 10 — Additional file 10. [file 12870_2020_2821_MOESM10_ESM.doc]

**The raw data of hormone detection**

| **Hormones** | **Line** | **Rep1** | **Rep2** | **Rep3** |
| --- | --- | --- | --- | --- |
| 0 h | WT | 1.20 | 0.42 | 1.14 |
|  | 11# | 3.18 | 1.29 | 2.84 |
| JA 72 h | WT | 2.00 | 2.04 | 1.87 |
|  | 11# | 3.23 | 2.49 | 5.86 |
| 120 h | WT | 9.52 | 2.62 | 2.60 |
|  | 11# | 4.77 | 15.39 | 6.99 |
|  | **Line** | **Rep1** | **Rep2** | **Rep3** |
| 0 h | WT | 0.15 | 0.13 | 0.14 |
|  | 11# | 0.28 | 0.30 | 0.19 |
| JA-Ile 72 h | WT | 0.17 | 0.20 | 0.15 |
|  | 11# | 0.22 | 0.24 | 0.99 |
| 120 h | WT | 0.32 | 0.14 | 0.16 |
|  | 11# | 0.74 | 0.93 | 0.57 |

**The raw data of disease index**

| **Metabolites** | **Repetions** | | |
| --- | --- | --- | --- |
| **Galactol** | **Rep1** | **Rep2** | **Rep3** |
| CK | 1.633 | 1.844 | 1.739 |
| 0.1mM | 1.173 | 1.189 | 1.181 |
| 0.5mM | 0.587 | 0.647 | 0.617 |
| 1mM | 0.846 | 0.950 | 0.898 |
| **Tyramine** | **Rep1** | **Rep2** | **Rep3** |
| CK | 1.633 | 1.844 | 1.739 |
| 1mM | 0.431 | 0.600 | 0.515 |
| 5mM | 0.745 | 0.693 | 0.719 |
| 10mM | 0.944 | 0.873 | 0.908 |
| **α-Terpinene** | **Rep1** | **Rep2** | **Rep3** |
| CK | 1.633 | 1.844 | 1.739 |
| 0.0007mM | 0.246 | 0.244 | 0.245 |
| 0.0025mM | 0.647 | 0.674 | 0.660 |
| 0.0040mM | 0.533 | 0.541 | 0.537 |
| **L-Glutamine** | **Rep1** | **Rep2** | **Rep3** |
| CK | 1.633 | 1.844 | 1.739 |
| 0.35mM | 0.291 | 0.418 | 0.355 |
| 0.7mM | 0.491 | 0.327 | 0.409 |
| 1.4mM | 0.760 | 0.782 | 0.771 |
| **L-Tryptophan** | **Rep1** | **Rep2** | **Rep3** |
| CK | 1.633 | 1.844 | 1.739 |
| 0.01mM | 0.500 | 0.386 | 0.443 |
| 0.1mM | 0.527 | 0.486 | 0.506 |
| 1mM | 0.629 | 0.536 | 0.592 |
| **Dopamine hydrochloride** | **Rep1** | **Rep2** | **Rep3** |
| CK | 1.633 | 1.844 | 1.739 |
| 0.001mM | 1.092 | 1.243 | 1.168 |
| 0.01mM | 0.587 | 0.700 | 0.665 |
| 0.1mM | 0.700 | 0.803 | 0.767 |

**The raw data of qRT-PCR.**

| **Gene** | **Ct** | | | |  |
| --- | --- | --- | --- | --- | --- |
| ***EF-1α*** | **Line** | **Rep1** | **Rep2** | **Rep3** |  |
| 0 h | WT | 21.49 | 21.47 | 21.31 |  |
|  | 11# | 21.29 | 21.2 | 21.38 |  |
| 72 h | WT | 20.55 | 20.89 | 20.58 |  |
|  | 11# | 21.04 | 21.37 | 21.2 |  |
| 120 h | WT | 21.19 | 21.03 | 21.18 |  |
|  | 11# | 20.15 | 20.14 | 20.13 |  |
| ***OsLOX1*** | **Line** | **Rep1** | **Rep2** | **Rep3** |  |
| 0 h | WT | 24.26 | 24.24 | 23.90 |  |
|  | 11# | 24.04 | 24.19 | 23.90 |  |
| 72 h | WT | 23.26 | 23.75 | 23.20 |  |
|  | 11# | 23.76 | 23.82 | 23.29 |  |
| 120 h | WT | 24.31 | 24.42 | 24.28 |  |
|  | 11# | 23.40 | 23.49 | 23.11 |  |
| ***OsLOX3*** | **Line** | **Rep1** | **Rep2** | **Rep3** |  |
| 0 h | WT | 34.31 | 34.77 | 34.54 |  |
|  | 11# | 30.75 | 30.40 | 31.09 |  |
| 72 h | WT | 29.82 | 30.06 | 30.47 |  |
|  | 11# | 24.40 | 24.06 | 24.59 |  |
| 120 h | WT | 28.92 | 28.60 | 29.50 |  |
|  | 11# | 24.35 | 24.07 | 24.58 |  |
| ***OsOPR1*** | **Line** | **Rep1** | **Rep2** | **Rep3** |  |
| 0 h | WT | 30.53 | 30.24 | 30.36 |  |
|  | 11# | 31.72 | 31.42 | 32.02 |  |
| 72 h | WT | 31.13 | 30.83 | 30.99 |  |
|  | 11# | 30.09 | 30.18 | 30.26 |  |
| 120 h | WT | 30.74 | 31.06 | 31.58 |  |
|  | 11# | 32.07 | 32.12 | 32.18 |  |
| ***OsOPR7*** | **Line** | **Rep1** | **Rep2** | **Rep3** |  |
| 0 h | WT | 24.97 | 24.95 | 24.90 |  |
|  | 11# | 25.03 | 25.21 | 25.39 |  |
| 72 h | WT | 24.86 | 24.91 | 25.07 |  |
|  | 11# | 24.98 | 25.06 | 25.29 |  |
| 120 h | WT | 25.31 | 25.30 | 25.42 |  |
|  | 11# | 25.05 | 25.12 | 25.05 |  |
| ***OsJMT1*** | **Line** | **Rep1** | **Rep2** | **Rep3** |  |
| 0 h | WT | 30.09 | 29.92 | 30.00 |  |
|  | 11# | 30.90 | 30.90 | 30.89 |  |
| 72 h | WT | 30.79 | 30.79 | 30.79 |  |
|  | 11# | 29.56 | 29.89 | 29.73 |  |
| 120 h | WT | 30.42 | 30.63 | 30.21 |  |
|  | 11# | 31.21 | 31.00 | 31.46 |  |
| ***OsHPL3*** | **Line** | **Rep1** | **Rep2** | **Rep3** |  |
| 0 h | WT | 30.19 | 29.45 | 29.07 |  |
|  | 11# | 28.78 | 31.19 | 31.63 |  |
| 72 h | WT | 28.56 | 27.36 | 27.84 |  |
|  | 11# | 25.35 | 27.97 | 28.37 |  |
| 120 h | WT | 30.06 | 30.04 | 29.71 |  |
|  | 11# | 29.12 | 30.28 | 30.32 |  |
| ***COI1b*** | **Line** | **Rep1** | **Rep2** | **Rep3** |  |
| 0 h | WT | 22.40 | 22.35 | 22.37 |  |
|  | 11# | 22.62 | 22.25 | 22.98 |  |
| 72 h | WT | 22.17 | 21.87 | 22.06 |  |
|  | 11# | 22.97 | 23.01 | 23.19 |  |
| 120 h | WT | 23.47 | 23.16 | 23.19 |  |
|  | 11# | 22.67 | 22.64 | 22.91 |  |
| ***OsJAZ1*** | **Line** | **Rep1** | **Rep2** | **Rep3** |  |
| 0 h | WT | 23.68 | 23.64 | 23.90 |  |
|  | 11# | 24.32 | 24.85 | 25.09 |  |
| 72 h | WT | 24.14 | 24.12 | 24.08 |  |
|  | 11# | 26.14 | 26.06 | 26.20 |  |
| 120 h | WT | 25.23 | 25.51 | 26.25 |  |
|  | 11# | 26.02 | 26.29 | 26.43 |  |
| ***OsJAZ9*** | **Line** | **Rep1** | **Rep2** | **Rep3** |  |
| 0 h | WT | 24.90 | 24.94 | 24.90 |  |
|  | 11# | 27.17 | 26.98 | 27.35 |  |
| 72 h | WT | 26.52 | 26.59 | 26.68 |  |
|  | 11# | 27.53 | 28.51 | 28.12 |  |
| 120 h | WT | 26.96 | 27.28 | 27.45 |  |
|  | 11# | 28.84 | 28.97 | 28.89 |  |
| ***OsMYC2*** | **Line** | **Rep1** | **Rep2** | **Rep3** |  |
| 0 h | WT | 21.69 | 21.49 | 21.44 |  |
|  | 11# | 22.66 | 23.04 | 22.85 |  |
| 72 h | WT | 22.59 | 22.04 | 22.21 |  |
|  | 11# | 23.96 | 24.44 | 24.09 |  |
| 120 h | WT | 24.38 | 23.76 | 23.69 |  |
|  | 11# | 24.20 | 24.58 | 24.11 |  |
| ***JiOsPR10*** | **Line** | **Rep1** | **Rep2** | **Rep3** |  |
| 0 h | WT | 25.13 | 25.25 | 25.19 |  |
|  | 11# | 23.42 | 23.27 | 23.58 |  |
| 72 h | WT | 24.42 | 24.63 | 24.80 |  |
|  | 11# | 26.28 | 26.22 | 26.24 |  |
| 120 h | WT | 25.25 | 25.40 | 25.37 |  |
|  | 11# | 25.41 | 25.57 | 25.48 |  |
| ***OsEDS1*** | **Line** | **Rep1** | **Rep2** | **Rep3** |  |
| 0 h | WT | 25.51 | 25.91 | 25.78 |  |
|  | 11# | 26.15 | 25.70 | 26.61 |  |
| 72 h | WT | 26.37 | 26.25 | 26.29 |  |
|  | 11# | 25.59 | 25.40 | 25.86 |  |
| 120 h | WT | 26.28 | 26.18 | 26.29 |  |
|  | 11# | 25.94 | 26.21 | 25.56 |  |
| ***OsPAD4*** | **Line** | **Rep1** | **Rep2** | **Rep3** |  |
| 0 h | WT | 24.72 | 24.91 | 24.91 |  |
|  | 11# | 25.84 | 25.64 | 26.04 |  |
| 72 h | WT | 26.38 | 26.30 | 26.21 |  |
|  | 11# | 24.15 | 24.11 | 24.43 |  |
| 120 h | WT | 26.22 | 26.13 | 26.24 |  |
|  | 11# | 26.18 | 26.50 | 26.26 |  |
| ***OsNPR4*** | **Line** | **Rep1** | **Rep2** | **Rep3** |  |
| 0 h | WT | 22.33 | 22.56 | 22.52 |  |
|  | 11# | 23.05 | 23.01 | 23.03 |  |
| 72 h | WT | 23.99 | 23.70 | 23.93 |  |
|  | 11# | 22.33 | 22.20 | 22.39 |  |
| 120 h | WT | 23.04 | 23.09 | 23.17 |  |
|  | 11# | 23.46 | 23.64 | 23.52 |  |
| ***OsOXO4*** | **Line** | **Rep1** | **Rep2** | **Rep3** |  |
| 0 h | WT | 26.84 | 26.75 | 27.02 |  |
|  | 11# | 29.28 | 29.18 | 29.38 |  |
| 72 h | WT | 26.16 | 26.40 | 26.11 |  |
|  | 11# | 24.31 | 24.31 | 24.10 |  |
| 120 h | WT | 26.15 | 26.27 | 26.25 |  |
|  | 11# | 25.95 | 26.01 | 25.79 |  |
| ***OsCHI11*** | **Line** | **Rep1** | **Rep2** | **Rep3** |  |
| 0 h | WT | 32.61 | 32.21 | 32.41 |  |
|  | 11# | 32.80 | 32.38 | 32.53 |  |
| 72 h | WT | 31.98 | 31.93 | 31.89 |  |
|  | 11# | 31.99 | 32.38 | 32.18 |  |
| 120 h | WT | 32.41 | 32.24 | 32.08 |  |
|  | 11# | 31.85 | 31.96 | 31.47 |  |
| ***OsPR10a*** | **Line** | **Rep1** | **Rep2** | **Rep3** |  |
| 0 h | WT | 25.20 | 25.34 | 25.40 |  |
|  | 11# | 22.35 | 22.07 | 22.63 |  |
| 72 h | WT | 21.72 | 21.72 | 21.91 |  |
|  | 11# | 24.53 | 24.83 | 24.94 |  |
| 120 h | WT | 21.97 | 21.94 | 22.24 |  |
|  | 11# | 22.24 | 22.57 | 22.63 |  |
| ***OsPR4a*** | **Line** | **Rep1** | **Rep2** | **Rep3** |  |
| 0 h | WT | 27.51 | 27.52 | 27.55 |  |
|  | 11# | 26.39 | 26.10 | 26.67 |  |
| 72 h | WT | 25.29 | 25.18 | 25.12 |  |
|  | 11# | 26.74 | 26.80 | 26.83 |  |
| 120 h | WT | 26.15 | 26.52 | 26.77 |  |
|  | 11# | 25.95 | 25.98 | 25.80 |  |
| ***OsPR5*** | **Line** | **Rep1** | **Rep2** | **Rep3** |  |
| 0 h | WT | 31.99 | 31.90 | 32.47 |  |
|  | 11# | 24.75 | 24.36 | 25.14 |  |
| 72 h | WT | 24.05 | 24.14 | 24.38 |  |
|  | 11# | 28.19 | 28.17 | 28.44 |  |
| 120 h | WT | 23.25 | 23.33 | 23.78 |  |
|  | 11# | 23.79 | 23.92 | 24.10 |  |
| ***OsPR8*** | **Line** | **Rep1** | **Rep2** | **Rep3** |  |
| 0 h | WT | 28.72 | 29.28 | 28.32 |  |
|  | 11# | 23.40 | 23.22 | 23.58 |  |
| 72 h | WT | 23.27 | 23.99 | 22.89 |  |
|  | 11# | 27.52 | 27.27 | 27.96 |  |
| 120 h | WT | 23.75 | 23.93 | 23.48 |  |
|  | 11# | 23.24 | 23.35 | 23.61 |  |
| ***EF-1α*** | **Line** | **Rep1** | **Rep2** | **Rep3** | **Rep4** |
| 0 h | WT | 21.97 | 21.85 | 22.15 | 22.14 |
|  | 11# | 21.90 | 22.02 | 21.42 | 21.85 |
| 72 h | WT | 22.31 | 22.04 | 22.27 | 22.61 |
|  | 11# | 22.06 | 22.06 | 21.97 | 21.94 |
| 120 h | WT | 21.37 | 21.50 | 21.42 | 21.48 |
|  | 11# | 21.13 | 21.36 | 21.44 | 21.45 |
| ***OsbHLH35*** | **Line** | **Rep1** | **Rep2** | **Rep3** | **Rep4** |
| 0 h | WT | 24.38 | 24.32 | 24.24 | 24.44 |
|  | 11# | 25.04 | 25.26 | 25.16 | 25.03 |
| 72 h | WT | 30.20 | 29.99 | 29.85 | 29.91 |
|  | 11# | 28.77 | 28.31 | 28.44 | 28.73 |
| 120 h | WT | 29.72 | 30.09 | 29.76 | 30.08 |
|  | 11# | 29.26 | 29.44 | 29.27 | 28.89 |
| ***Os01g0170000*** | **Line** | **Rep1** | **Rep2** | **Rep3** | **Rep4** |
| 0 h | WT | 25.91 | 25.79 | 25.82 | 25.99 |
|  | 11# | 26.87 | 26.69 | 26.52 | 26.73 |
| 72 h | WT | 27.16 | 27.11 | 27.09 | 27.27 |
|  | 11# | 27.72 | 27.61 | 27.40 | 27.54 |
| 120 h | WT | 27.95 | 28.15 | 28.14 | 28.21 |
|  | 11# | 27.93 | 27.89 | 27.97 | 27.91 |
| ***OsGolS1*** | **Line** | **Rep1** | **Rep2** | **Rep3** | **Rep4** |
| 0 h | WT | 23.86 | 23.89 | 23.50 | 23.82 |
|  | 11# | 23.99 | 23.89 | 23.81 | 24.08 |
| 72 h | WT | 24.19 | 23.78 | 23.94 | 24.11 |
|  | 11# | 24.03 | 23.73 | 23.82 | 24.21 |
| 120 h | WT | 25.11 | 25.30 | 25.49 | 25.45 |
|  | 11# | 24.52 | 24.51 | 24.65 | 24.56 |
| ***OsRSUS1*** | **Line** | **Rep1** | **Rep2** | **Rep3** | **Rep4** |
| 0 h | WT | 25.45 | 25.37 | 25.05 | 25.47 |
|  | 11# | 27.01 | 26.92 | 26.55 | 27.02 |
| 72 h | WT | 26.39 | 27.24 | 27.76 | 28.23 |
|  | 11# | 27.61 | 27.32 | 27.48 | 27.44 |
| 120 h | WT | 26.34 | 26.29 | 26.41 | 26.78 |
|  | 11# | 26.33 | 26.45 | 26.61 | 26.81 |
| ***OsFRK-2*** | **Line** | **Rep1** | **Rep2** | **Rep3** | **Rep4** |
| 0 h | WT | 25.63 | 25.29 | 25.40 | 25.61 |
|  | 11# | 25.72 | 25.83 | 25.55 | 25.77 |
| 72 h | WT | 25.91 | 26.30 | 26.25 | 26.15 |
|  | 11# | 26.32 | 26.29 | 26.41 | 26.24 |
| 120 h | WT | 25.98 | 25.83 | 25.98 | 26.14 |
|  | 11# | 25.96 | 25.79 | 25.94 | 26.14 |
| ***OsWAK85*** | **Line** | **Rep1** | **Rep2** | **Rep3** | **Rep4** |
| 0 h | WT | 27.54 | 27.37 | 27.54 | 27.83 |
|  | 11# | 26.92 | 26.92 | 26.39 | 27.02 |
| 72 h | WT | 26.14 | 27.14 | 27.80 | 28.03 |
|  | 11# | 26.72 | 26.60 | 26.70 | 26.45 |
| 120 h | WT | 27.02 | 27.54 | 27.27 | 26.83 |
|  | 11# | 26.78 | 26.84 | 27.03 | 27.23 |
